# Head and Neck Mycetoma: The Mycetoma Research Centre Experience

**DOI:** 10.1371/journal.pntd.0003587

**Published:** 2015-03-13

**Authors:** Ahmed Fahal, EL Sheikh Mahgoub, Ahmed Mohamed EL Hassan, Angom Osman Jacoub, Doaa Hassan

**Affiliations:** The Mycetoma Research Centre, University of Khartoum, Khartoum, Sudan; University of California San Diego School of Medicine, UNITED STATES

## Abstract

Mycetoma is a unique neglected tropical disease which is endemic in what is known as the “mycetoma belt”. The disease has many devastating impacts on patients and communities in endemic area and is characterised by massive deformity, destruction and disability. Mycetoma is commonly seen in the foot and hand and less frequent in other parts of the body. Mycetoma of the head and neck is a rarity and is associated with high morbidity and even mortality if not treated early. In this communication we report on 49 patients with head and neck mycetoma followed up at the Mycetoma Research Centre in Khartoum. Most of the reported patients had actinomycetoma and the majority were young adult males from mycetoma endemic areas in the Sudan. Most of them were students, farmers and workers. Prior to presentation the majority had long disease duration and the cause was multifactorial. Advanced disease with massive lesion, deformity and disability was the common presentation. There was no obvious history of local trauma, familial tendency or other predisposing factor identified in this group of patients. MRI and CT scan were the most accurate diagnostic tools to determine the disease extent. The treatment outcome was rather poor and characterised by low cure rate, poor outcome and high follows-up dropout. Such a gloomy outcome calls for structured and objective health education programs.

## Author Summary

Although head and neck mycetoma is a rare disease entity yet it is a dreadful disease for the patient, the family and the treating physician. It is potentially fatal and the most challenging to treat. The current study highlighted that, most patients were young adult males, from rural areas of the Sudan and of low socioeconomic status. The lack of medical and health facilities, financial constrains and compounded by poor health education in endemic areas meant that most of the studied patients presented late with advanced disease. The diagnosis of mycetoma in the studied population was confirmed by several imaging techniques; MRI, CT scan and radiography and tissue diagnosis by histopathology and cytology techniques. The treatment outcome was rather unsatisfactory. The cure rate was low, the dropout rate was high and the disease was associated with high morbidity. Structured and objective health education programmes in the endemic areas is important to encourage patients to seek medical advice early in the course of the disease particularly that there is no clear preventive or control measurement in mycetoma.

## Introduction

Mycetoma is one of the neglected tropical diseases, characterised by massive deformity, disability and can be fatal if not managed properly and timely [1–3]. It is a chronic, specific, granulomatous, progressive subcutaneous inflammatory disease that spreads to involve the skin, deep structures and bones [4,5]. The disease is caused by true fungi or by certain bacteria and hence it is usually classified into eumycetoma and actinomycetoma, respectively [6,7]. *Madurella mycetomatis* is the commonest eumycetoma causative agent while *Streptomyces somaliensis* and *Nocardiae* are the common causative organisms for actinomycetoma [8–10]. Mycetoma has a definite geographic distribution and it is endemic in what is known as the “Mycetoma Belt” that includes Sudan, Senegal, Somalia, South India, South America and Mexico; however, it is reported in many other countries [11–17]. The infection usually progresses slowly over many years and it is commonly painless and that may contribute to the late presentation of many patients [2,18]. The painless subcutaneous mass, multiple sinuses and discharge with grains is distinctive of this infection [1] Young adult males in the age range 20–40 years are more frequently affected [2,4]. Farmers, workers and students are affected most but no occupation is exempted [2,5].

The diagnosis of mycetoma is tedious and several tools are required to reach a proper diagnosis. These tools include imaging techniques such as radiography, ultrasonography, CT, MRI [19–21], molecular techniques such as PCR [22], serodiagnosis as ELISA, CIE [23, 24] as well the classical grain culture and histopathological diagnosis [23]. Although the disease can be diagnosed clinically this is not accurate and can be misleading.

Early lesions are amenable to medical and surgical treatment with good prognosis [24,25]. Generally, actinomycetoma responds to medical treatment in the form of combined antibiotics while eumycetoma requires both antifungal and surgical excision [26,27]. Late and advanced disease is difficult to treat, has poor prognosis and is associated with high recurrence and amputation rates [28]. Currently there is no preventive or control measurements as the route of infection, susceptibility and resistance to the infection are still an enigma and hence health education is essential to avoid the disease and its high morbidity and complications. Mycetoma of the head and neck region is a rarity, patients commonly present with massive lesions and is associated with poor prognosis and can be fatal. In this communication, the Mycetoma Research Centre of the University of Khartoum experience of managing 49 patients with mycetoma of the head and neck region is presented.

## Materials and Methods

This descriptive, cross-sectional hospital based study was conducted at the Mycetoma Research Centre (MRC), University of Khartoum, Khartoum, Sudan. The study included 49 patients with confirmed head and neck mycetoma seen in the period January 1991 and October 2014. The diagnosis of mycetoma was confirmed by careful interview, meticulous clinical examinations and standard investigations.

The investigations included fine needle aspiration for cytology (FNA), histopathological examination of surgical biopsies using different staining techniques and grains culture in various media. The common sero-diagnostic test used was counter-immuno-electrophoresis. Different imaging techniques were used and that included radiography of the affected sites in at least in two views: anterio-posterior and lateral, lesion ultrasound examination, and in some patients MRI and CT scan. The electronic patients’ notes were carefully and meticulously reviewed.

### Ethical Statement

The study ethical clearance was obtained from Soba University Hospital Ethical Committee, it waived the need for consent.

### Statistical Analysis

Statistical analysis was conducted using SPSS computer programme. Data was summarized as percentages for categorical variables and mean ± standard error of the mean (SEM) and median for continuous variables.

## Results

The 49 studied patients with confirmed head & neck mycetoma constituted 0.76% of the total MRC patient population seen during the study duration. In the present study, 33 patients (67.3%) had actinomycetoma and 16 (32.7%) had eumycetoma. There were 39 males (79.6%) and 10 females (20.4%). Their ages ranged between 9 and 67 years with a mean age of 27.9 ± 14.7 years. 31(63.3%) of the patients were under 40 years-old at presentation and 23(46.9%) were in the age group 1–20 years. Only five patients (10.2%) were more than 50 years of age at presentation.

In this study, there were 14 students (28.6%), 11 workers (22.4%) and 10 farmers (20.4%). Due to prolonged illness and disability, seven patients (14.3%) were unemployed. There were five housewives (10.2%) in this population.

The majority of the patients, 27(55.1%) were from central Sudan; AL Jazeera State 10(20.4%) and Sinnar State 7(14.3%). There were eight patients (16.3%) from Kordofan States, seven patients (14.3%) from Khartoum State, five patients (10.2%) from Kassala State, four patients (8.1%) form Darfour States and three patients (6.1%) from the White Nile State.

The disease duration at presentation ranged between one and 40 years with a mean duration of 11.23 ± 19.7 years. The majority of the patients 33(67.3%) had mycetoma for less than 10 year and 16(32.7%) of them had the disease for less than one year. Only three patients (6.1%) had the disease for more than 30 years.

Thirty patients (61.2%) had history of discharge contained grains and the colour of the grains was yellow (38.8%), black (24.5%) or white (8.2%). Pain at the mycetoma site, was not a frequent symptom among the study population; documented in only 11 patients (22.4%). Only fourteen patients (28.6%), recalled history of local trauma at the mycetoma site and three patients could not recall such a history. Concomitant other illness was documented in only two patients (4.1%). Four patients (8.2%) had family history of mycetoma.

The majority of patients, 36(73.5%) had recurrent disease and underwent previous surgical excisions; 23 patients (46.9%) had one surgical excision, five patients (10.2%) underwent two surgical excisions, six patients (12.2%) had three previous surgical excisions while two patients (4.1%) had more than three surgical excisions. The type of anaesthesia used ranged between general (41.8%) and local (58.2%).

**Fig 1:**
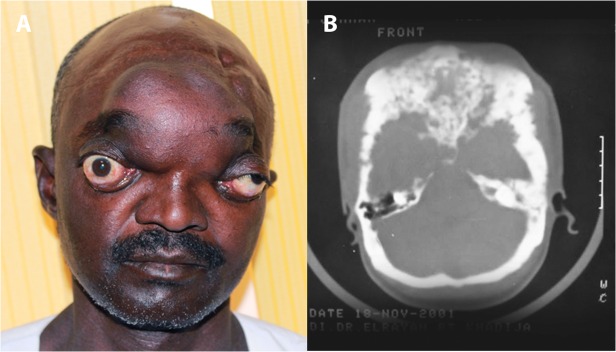
A: Showing massive head actinomycetoma with severe bilateral proptosis. **This patient had vision loss in the right eye, and loss of hearing in both ears due massive intra-cranial involvement.** B: Skull CT scan showing massive intracranial actinomycetoma involvement with bones destruction. The patient agreed to show his photos for publication purpose.

**Fig 2:**
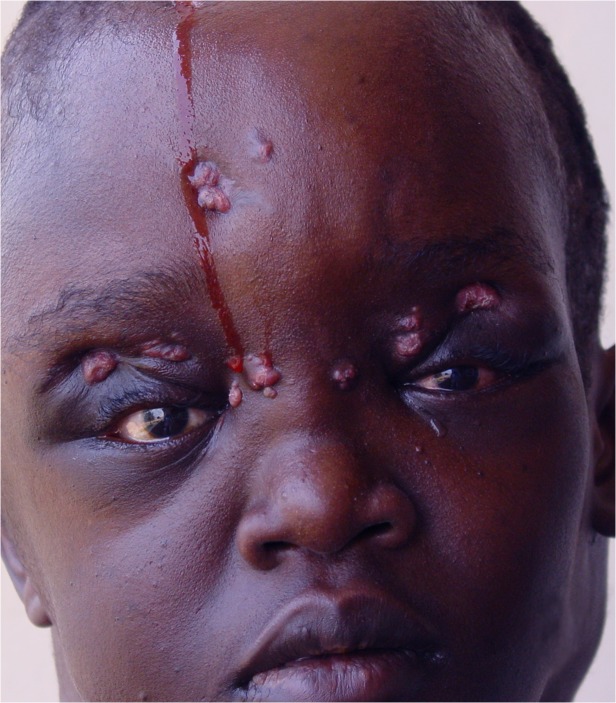
Showing massive head actinomycetoma with involvement of the different parts of the skull with multiple sinuses and discharge. The patient agreed to show her photos for publication purpose.

**Fig 3:**
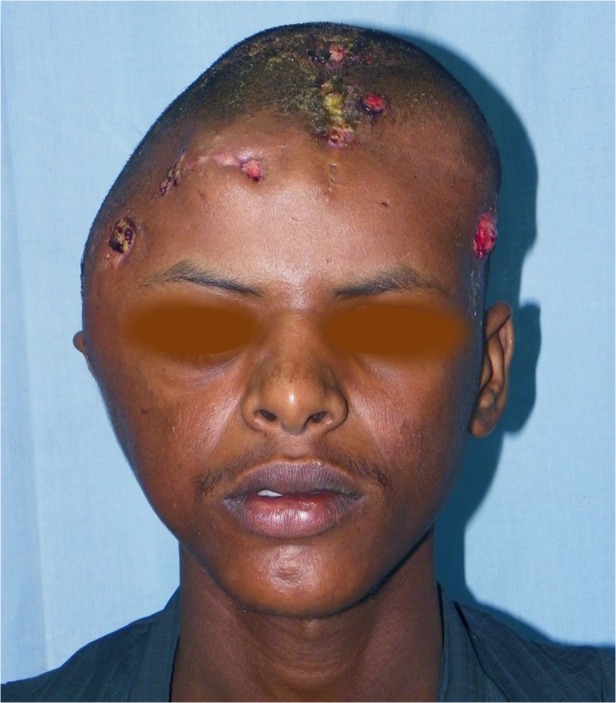
Showing massive head actinomycetoma with multiple sinuses. The patient agreed to show his photos for publication purpose.

Different parts of the head and neck were involved which included the frontal (n = 12), occipital (n = 5), parietal (n = 1) and temporal region (n = 1). Multiple skull bones involvement was documented in 12 patients, (Table 1). Five patients had combined frontal and parietal and/ or temporal bone involvement. Two patients had combined occipito-temporo-parietal bones affection. One patient had massive sphenoid, ethmoid, maxillary, nasal bones, anterior cranial fossa, temporal, frontal and occipital bones and supra-orbital areas. One patient had infra-temporal fossa mycetoma extending to the nasopharynx involvement. Two patients had base of the skull and occipital mycetoma with cervical region extension. The orbit was involved in two patients. The upper eye lid, buccal cavity and cheek were affected in one each. Ten patients (20%) had cervical mycetoma. Four patients (4%) had intracranial lesions (Figs. 1, 2, 3). 

**Table 1:** Table showing the head and neck distribution of mycetoma among the studied population.

Site	No.	%
**Frontal region**	**12**	**24.5**
**Occipital region**	**5**	**10.2**
**Temporal region**	**1**	**2.0**
**Parietal region**	**1**	**2.0**
**Cheek**	**2**	**4.0**
**Orbit**	**2**	**4.0**
**Buccal cavity**	**1**	**2.0**
**Upper eye lid**	**1**	**2.0**
**Fronto-parietal regions**	**2**	**4.0**
**Fronto-temporo-parietal regions**	**2**	**4.0**
**Fronto-temporal regions**	**1**	**2.0**
**Frontal bone and supra-ocular region with parietal extension**	**1**	**2.0**
**Occipito-parietal regions**	**1**	**2.0**
**Occipito-temporo-parietal regions**	**1**	**2.0**
**Sphenoid, ethmoid, maxillary, nasal bones, anterior cranial fossa, with temporal, frontal and occipital regions and supra-orbital areas**	**1**	**2.0**
**Infra-temporal fossa extending to the naso-pharynx**	**1**	**2.0**
**Skull base and neck involvement**	**1**	**2.0**
**Occipital with upper cervical bone involvement**	**1**	**2.0**
**Neck only**	**10**	**20.4**

The mycetoma lesions were classified according to their sizes into small (less than 5 cm), moderate lesion (5–10 cm) and massive lesion (>10cm). The study showed that, 20 patients (40.8%) had massive lesions at presentation while 12 patients (24.5%) had small lesions. At presentation, 30 patients (61.2%) had lesions with sinuses; they were active in 15 patients (30.6%), healed in six patients (12.2%) and nine patients (18.3%) had both active and healed sinuses.

Grains discharged through the sinuses were not detected on clinical examination in 37 patients (75.5%) while in 12 patients (24.5%) grains were detected. Local hyper-hydrosis at and around the mycetoma lesion was detected in one patient (2%).

Regional lymph nodes enlargement was detected in six patients (12.2%). Dilated tortuous veins proximal to the mycetoma lesions were not detected in the present. One patient presented with massive intracranial eumycetoma with minimal skin and subcutaneous affection.

**Fig 4:**
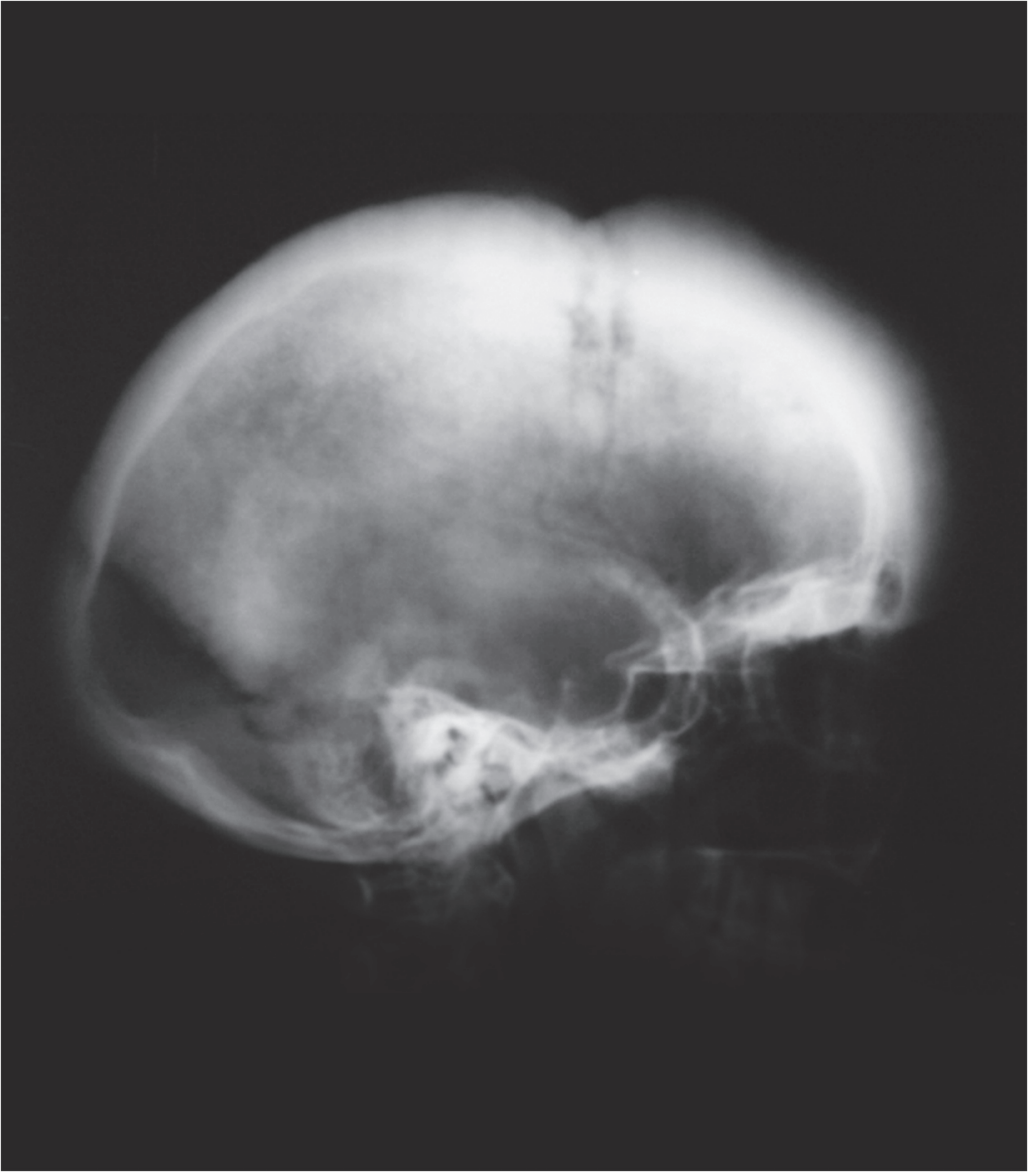
Skull X-ray showing massive thickening of the skull tables and generalised osteoblastic changes.

At presentation 30 patients had skull and cervical X-Ray examination in at least two views and that showed normal findings in eight patients (16.3%), soft tissue mass in 10(20.4%), periosteal reaction in one patient (2%), bone destruction in five patients (10.2%) and in six patients (12.2%) a combination of these findings were detected (Fig. 4).

Ultrasound examination of the mycetoma lesion was performed in 11 patients (22.4%). This showed evidence of eumycetoma in five patients (10.2%), actinomycetoma in four patients (8.2%) while in two patients (4.1%) no diagnosis was established.

**Fig 5:**
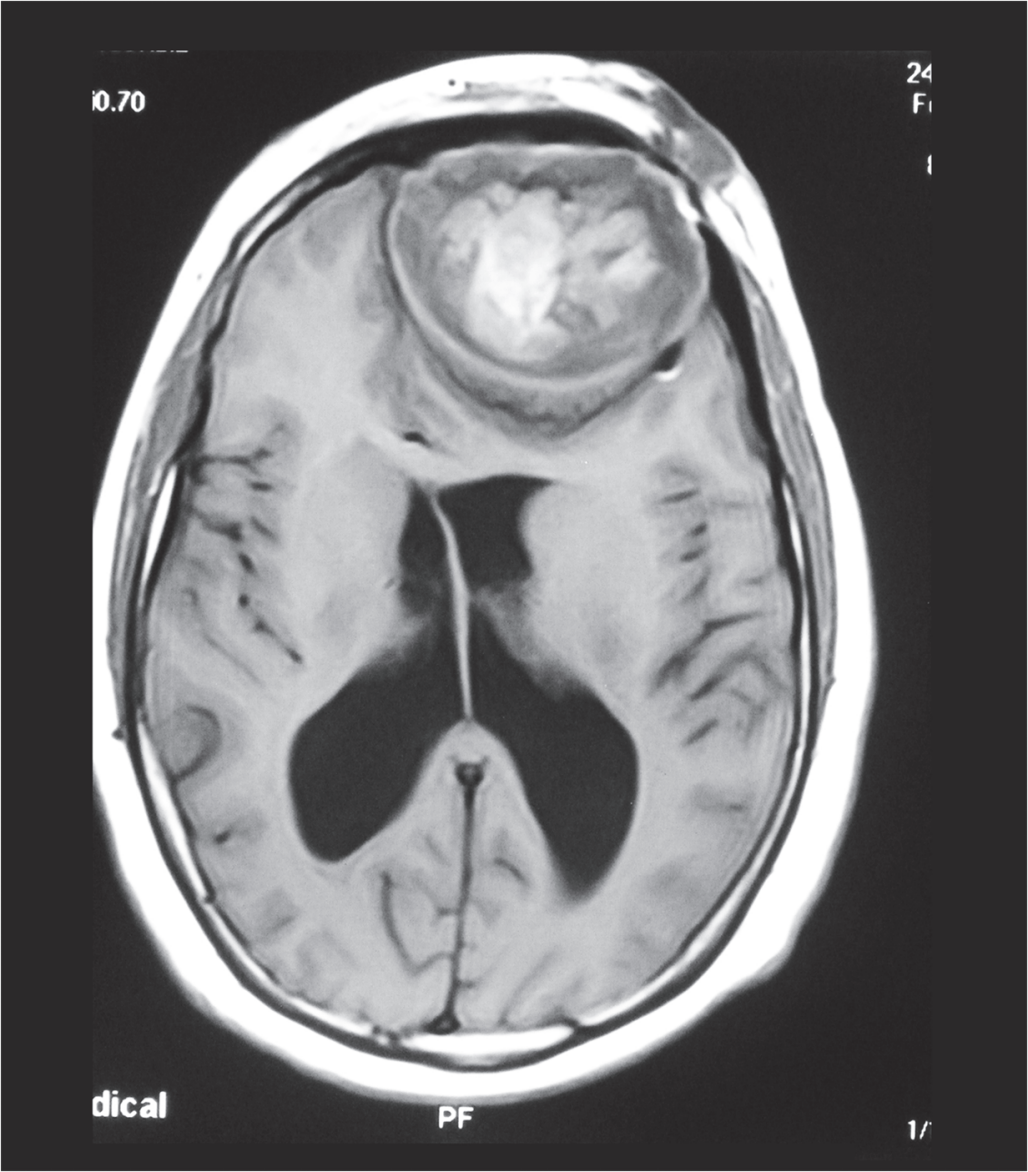
Cranial MRI showing eumycetoma involving the skin, subcutaneous tissue and destruction of the frontal bone and with well encapsulated intracranial lesion.

**Fig 6:**
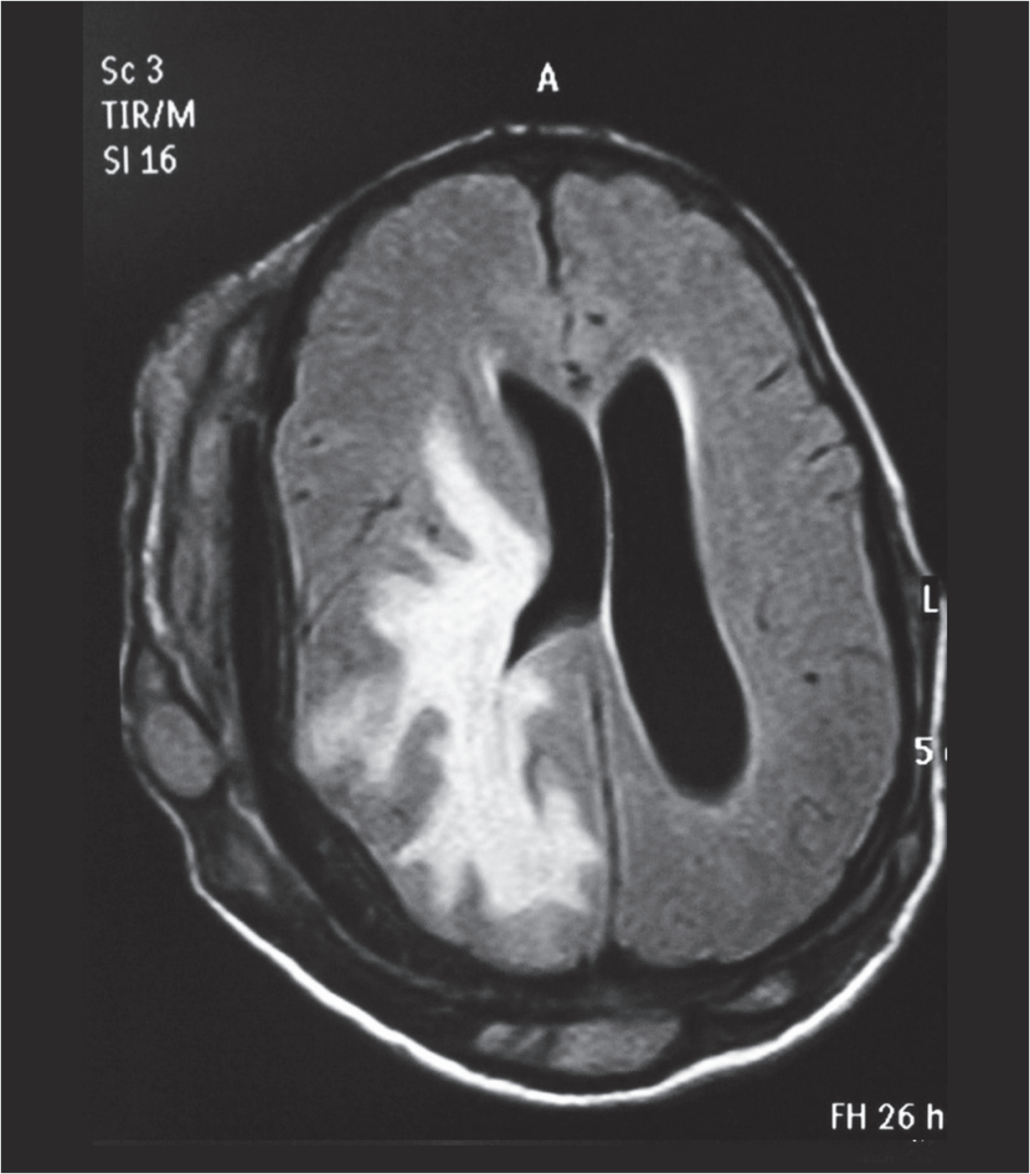
Cranial MRI showing generalised skin, subcutaneous tissue and bone involvement with massive intracranial spread.

Most of the patients had MRI examination and it showed the skin, subcutaneous, skull and intracranial disease spread with the typical dot-in-circle sign in most of them, (Figs. 5, 6).

FNA for cytology was performed in 21 patients to confirm the diagnosis and it showed evidence of *Actinomadura madurae* in eight patients (16.3%), *M*. *mycetomatis* in six patients (12.2%), *Streptomyces somaliensis* in four patients (8.1%) and in three patients (6.1%) no grains were not detected.

In this series, 24 patients (49%) had histopathological examinations of surgical biopsies. The diagnosis of *M*. *mycetomatis* was established in nine patients (18.4%), *Streptomyces somaliensis* in 11 patients (22.4%) and *Actinomadura madurae* in three patients (6.1%). In one patient (2%) no diagnosis was established due to grains absence and the diagnosis was established by FNA.

For actinomycetoma a combination of antimicrobial agents was given and that included streptomycin sulphate and dapsone, or streptomycin and trimethoprim-sulfamethoxazole. More recently, trimethoprim-sulfamethoxazole 8/40 mg/kg/day in cycles for 5 weeks and amikacin 15 mg/kg/day in a divided dose every 12 hours for 3 weeks were administered. The two week interval of amikacin in the five-week cycle is used for renal and audiometric monitoring.

For eumycetoma several antifungal agents combined with various surgical excisions were performed. The common antifungal agents used were ketoconazole and Itraconazole.

All patients were offered follow up appointments but due to various reasons 14 patients (28.5%) were subsequently lost for follow up, five patients (10.2%) were completely cured, and 30 patients (61.2%) had partial cure.

## Discussion

The incidence of mycetoma of the head and neck region is infrequent. Review of the medical literature revealed only few reports on mycetoma in this site [10,29–31], and although Sudan is considered the mycetoma homeland, only few reports on head and neck mycetoma were reported. Lynch in 1964, reported on 1860 mycetoma patients and of these only 18 patients (0.96%) had head and neck mycetoma [15]. Mahgoub in 1977 reported an incidence of 3% of head and neck mycetoma [32]. In 1986, Gumaa and her associates reported on 15 out 400 patients with mycetoma (3.75%) involving the head and neck region. This communication is in line with the fact that, mycetoma at this region is a rarity.

In agreement with the previously reported series, actinomycetoma was the prevalent type of mycetoma in our series and the explanation for this prevalence remains unclear [10,29]. It is possible that the actinomycetes are resilient and able to survive in the extra-paedal areas more than eumyceteces.

Males were predominantly affected in our series and this is in accordance with previous reports from the Sudan [10,11,29]. Again the explanation for this is unclear; however there is suggestion that sex hormones play a role in this predominance [33]. The majority of the reported patients were young adults with a mean age of 27.9 ± 14.7 years and this is a typical age in mycetoma patients [4,10,34]. Students were affected most, and this may be explained by the fact that, young age groups of patients contract the disease more. The study showed that 44.8% of the affected patients were farmers and workers. This is an important finding as the nature of their work puts them in direct contact with the soil on a daily basis and it has been postulated that the soil harbours the causative organisms and these patients are constantly exposed to minor injuries which facilitate the traumatic subcutaneous inoculation of the organisms.

The mean disease duration at presentation among the affected study population is quite long. This may be explained by the painless nature of the disease, the lack of health education, low socio-economic status of the affected patients and lack of medical and health facilities in the endemic regions.

The clinical presentation of patients in this series was typical and in agreement with other reports [1,2,35]. It started gradually at the subcutaneous tissue and progressed to affect the deep structures. It was painless in the majority of patients and that may be an important contributory factor for the late presentation in most patients.

The study showed that 73.5% of the patients had multiple surgical excisions and recurrence and most of them had surgery performed under local anaesthesia. It is well known that incomplete surgical excision performed under local anaesthesia is the major factor leading in recurrence.

At presentation almost half of the patients had massive lesions which is caused by their late presentation and the fact that, most of them had actinomycetoma which is known to be aggressive and can invade the deep structures and bone at an early disease stage [10].

Different skull parts were affected in our series, however, the frontal and occipital parts were affected most. The explanation for this is unclear however these parts are more prone to direct trauma and hence local inoculation of the causative organisms. Rare sites were encountered and this included the eye.

One patient presented with massive intracranial eumycetoma with minimal skin and subcutaneous involvement, again the explanation is unclear but deep inoculation of the infection may provide some explanation.

In the past, the disease extends in the head and neck area was assessed clinically and radiologically by skull and cervical X-rays or by cerebral angiography which is invasive and with many complications. Currently, the use of the MRI and CT scans provided an accurate assessment with minimal complications. Mycetoma has characteristic MRI features which are diagnostic. The MRI can delineate the involvement of the skin, subcutaneous, muscles and bones accurately and can grade the disease and help in planning patients’ management [21].

The present series showed poor treatment outcome, only five patients were cured and this is in line with previous reports [28–30,36]. This low cure rate necessitates the need for more efficient and safe novel drugs for the treatment of mycetoma. The dropout rate (28.5%) in our series is high. The reasons for the high dropout rate are multifactorial and to mention but a few, the patients’ dissatisfaction due to the high cost and the prolonged treatment duration which is commonly more than one year duration, the drug side effects and complications, the patients low socio-economic status, the lack of health education and difficulty to reach the MRC, particularly during rainy seasons. All these can contribute to the poor treatment outcome.

In conclusion, mycetoma of the head and neck region is a serious medical and health problem, is associated with serious complications, low cure rate and high follow-up dropout rate. The route of infection, susceptibility and resistance in mycetoma remains poorly understood and this is compounded by the lack of preventive and control measures. Hence health education may be the only tool to reduce the disease morbidity and mortality.

## Supporting Information

S1 Checklist STROBE checklist. The patients agreed to show their photos for publication purpose.
(DOC)
